# Personalised Nutrition Approaches in the Prevention and Management of Type 2 Diabetes: A Narrative Review of Evidence and Practice

**DOI:** 10.3390/ijerph22071047

**Published:** 2025-06-30

**Authors:** Mabitsela Mphasha, Tebogo Mothiba

**Affiliations:** 1 Department of Public Health and Health Systems, University of Limpopo, Polokwane 0727, South Africa; 2 Deputy Vice Chancelor Research, Innovation and Partnerships, University of Limpopo, Polokwane 0700, South Africa; tebogo.mothiba@ul.ac.za

**Keywords:** type 2 diabetes mellitus, personalised nutrition, glycaemic index, glycaemic load, food insulin index, glycaemic control, precision nutrition, dietary intervention

## Abstract

Type 2 diabetes mellitus (T2DM) remains a significant global public health concern, largely driven by poor dietary habits, physical inactivity, and rising obesity rates. In recent years, personalised nutrition (PN) emerged as a promising approach to T2DM prevention and management. This narrative review synthesises current evidence on tailored dietary strategies, including the glycaemic index (GI), glycaemic load (GL), food insulin index (FII), and precision nutrition tools. It further explores their impact on glycaemic control, insulin sensitivity, and adherence to dietary interventions. A structured review of peer-reviewed and grey literature was conducted, taking into account behavioural, cultural, and systemic implementation factors. Although evidence supports the efficacy of PN in improving metabolic outcomes, implementation in low- and middle-income countries (LMICs) remains limited due to infrastructural, financial, and contextual challenges. This review emphasises the need for context-specific, scalable solutions integrated into primary healthcare systems.

## 1. Introduction

Type 2 diabetes mellitus (T2DM) is a major non-communicable disease (NCD) that poses a growing global public health challenge. It is characterised by chronic hyperglycaemia resulting from insulin resistance and/or impaired insulin secretion. The International Diabetes Federation (IDF) [1] reports that as of 2025, more than 589 million adults between the ages of 20 and 79 are living with diabetes, with projections estimating an increase to 853 million by the year 2050. Alarmingly, one in eight adults is at risk of developing T2DM, while over 252 million remain unaware of their condition. Notably, nearly three-quarters of the global diabetic population reside in low- and middle-income countries (LMICs), underscoring the urgent need for scalable, contextually appropriate, and accessible intervention strategies [1]. The sharp rise in T2DM incidence is largely attributable to modifiable risk factors, including unhealthy dietary habits, physical inactivity, and increasing rates of overweight and obesity [2]. These risk factors are further exacerbated in LMICs, particularly in sub-Saharan Africa, by rapid urbanisation, the nutrition transition from traditional to highly processed foods, and socio-economic barriers to healthcare access [3,4]. In many communities, healthcare services are overburdened and underfunded, with a stronger focus on infectious diseases and maternal–child health, leaving chronic conditions such as T2DM underprioritised. Moreover, the integration of nutrition into primary healthcare remains limited, undermining preventive efforts.

Historically, the dietary management of T2DM centred on generalised recommendations aimed at reducing energy intake, limiting saturated fats and added sugars, and increasing fibre and whole grains [5]. While these guidelines demonstrated some effectiveness at the population level, they often fail to consider the substantial inter-individual variability in metabolic responses to food. Factors such as genetics, gut microbiota composition, behavioural patterns, cultural food practices, and socio-economic status significantly influence dietary behaviours and outcomes. Consequently, the “one-size-fits-all” approach to dietary guidance may not be sufficient to ensure long-term adherence or optimise glycaemic control across diverse populations [6].

In response to these limitations, personalised nutrition (PN) gained momentum as a tailored approach to dietary intervention. PN refers to the customization of nutrition advice based on individual characteristics such as genetic profile, metabolic markers, lifestyle behaviours, cultural background, and personal preferences [7]. In this review, PN refers to tailoring dietary advice to an individual’s biology, lifestyle, and preferences to support type 2 diabetes prevention and management. The goal is to enhance engagement, dietary adherence, and metabolic outcomes through more precise and relevant nutritional strategies. Tools such as the glycaemic index (GI), glycaemic load (GL), food insulin index (FII), nutrigenomics, and microbiome profiling are increasingly used to inform personalised dietary planning [8,9]. For instance, individuals may exhibit significantly different glycaemic responses to the same carbohydrate-containing meal, underscoring the value of personalised dietary strategies. Moreover, the integration of digital health tools, such as mobile applications, wearable devices, and continuous glucose monitors (CGMs), further expanded the potential reach and impact of PN.

Although digital health technologies show significant potential to enhance adherence and engagement, especially among younger or technologically inclined populations [10], the adoption of PN in LMICs remains constrained. Key obstacles include insufficient healthcare infrastructure, a shortage of trained professionals, the high cost of diagnostic tools, and the absence of culturally appropriate dietary models [11,12]. These limitations underscore the urgent need for scalable and contextually relevant PN interventions that can be effectively integrated into primary healthcare systems.

This narrative review aims to synthesise the existing evidence on the use of personalised nutrition in the prevention and management of T2DM. It explores the effectiveness of various PN strategies, identifies implementation challenges, especially in LMICs, and discusses implications for clinical practice and health policy. By addressing both scientific evidence and practical considerations, this review seeks to inform the development of more inclusive, culturally sensitive, and sustainable approaches to diabetes care.

## 2. Methodology

This narrative review explores the effectiveness of personalised nutrition strategies for preventing and managing T2DM. Narrative reviews are suitable for examining complex topics. They allow the inclusion of various study designs and existing reviews into an integrated and interpretive synthesis. A structured and transparent method was followed to enhance the review’s rigour and credibility.

### 2.1. Literature Search Strategy

A comprehensive literature search was conducted without applying a publication year limit, given the relevance and evolving nature of the topic. The search included three major electronic databases: PubMed, Scopus, and Web of Science. To broaden the scope and ensure inclusion of practice-based evidence, grey literature, such as policy briefs, technical reports, and clinical guidelines, was also reviewed. These were retrieved from reputable organizations, including the World Health Organization (WHO) and the International Diabetes Federation (IDF).

The search strategy combined Medical Subject Headings (MeSH) and free-text keywords, refined using Boolean operators (AND, OR). The primary search terms included the following:

- “Personalized nutrition” OR “individualised dietary intervention”
- “Type 2 diabetes” OR “T2DM”
- “Glycaemic index” OR “GI”
- “Glycaemic load” OR “GL”
- “Food insulin index” OR “FII”.

These terms were used in various combinations to ensure comprehensive coverage of the literature.

### 2.2. Inclusion and Exclusion Criteria

Although formal quality appraisal tools were not used, predefined inclusion and exclusion criteria were applied to enhance consistency and transparency.

Inclusion criteria:

- Publications from published in English from 2008 to 2025.
- Peer-reviewed articles including clinical trials (RCTs), reviews, as well as quantitative and qualitative studies, including book chapters.
- Grey literature and policy documents relevant to dietary strategies in diabetes prevention or management.
- Studies focusing on glycaemic control, behavioural adherence, and personalised nutrition interventions.

Exclusion criteria:

- Studies not focused on T2DM.
- Articles lacking dietary or nutritional intervention focus.
- Letters to the editor, editorials, and conference abstracts with insufficient data.
- Non-English language publications.

### 2.3. Study Selection Process

The initial search yielded 1003 articles. After removing duplicates and screening titles and abstracts, 311 articles remained for full-text review. Of these, 58 met the inclusion criteria and were included in the final synthesis. The study selection process followed an informal, yet systematic screening process performed by the primary author, in consultation with the co-author.

### 2.4. Integration of Grey Literature

Grey literature was assessed based on the reputation and authority of the publishing institution (e.g., WHO and IDF). These sources were included to provide context on the scalability, feasibility, and real-world implementation of personalised nutrition strategies, especially in low-resource settings where peer-reviewed data may be limited.

### 2.5. Questions

The review was guided by the following overarching questions:

- What role does personalised nutrition play in preventing and managing T2DM?
- How do GI, GL, and FII-based strategies affect glycaemic control?
- Which behavioural, cultural, or systemic factors influence adherence?
- What are the challenges and facilitators of implementing these strategies in low-resource settings?

## 3. Nutrition and Its Role in Managing T2DM

Nutrition is a cornerstone of T2DM prevention and management. Evidence supports dietary measures such as reduced carbohydrate intake, increased fibre, and the use of low-GI foods in improving glycaemic control and reducing the risk of complications [13]. In early-stage T2DM, lifestyle interventions can be as effective as pharmacological treatment in achieving glycaemic targets [14,15]. Even modest weight loss (5–10% of body weight) significantly enhances insulin sensitivity and may induce remission in some individuals [16].

Nonetheless, responses to similar dietary interventions vary widely between individuals. Factors such as genetic predisposition, gut microbiota composition, insulin sensitivity, and behavioural habits contribute to this variability [17,18]. These inter-individual differences led to growing support for personalised nutrition, an approach that tailors’ dietary recommendations to an individual’s metabolic profile, lifestyle, and food preferences. Tools including GL and the FII offer added precision for dietary planning and self-management, especially when incorporated into structured education [19]. In culturally diverse settings, these strategies become even more impactful when adapted to align with local food practices and socio-cultural norms. Tailored nutrition has the potential to improve adherence, empower patients, and deliver sustainable outcomes in T2DM care.

## 4. Personalised Nutrition in T2DM: Strategies and Evidence

- **Glycaemic index, glycaemic load, and food insulin index (FII):** Low-GI and low-GL diets reduce postprandial glucose excursions and improve HbA1c and insulin sensitivity [8,20]. These measures have long been considered useful in guiding dietary choices for glycaemic control. While GL offers better predictive power than GI alone, FII remains underutilised due to limited data and clinical guidance. The FII captures the insulin response to all macronutrients, offering a more holistic assessment of dietary impact [9]. However, the FII clinical application is limited due to a lack of global standardisation, a narrow range of tested foods, exclusion from major nutrient databases, and unclear guidelines for practical use.
- **Genetic and metabolic profiling:** Genetic and metabolic profiling: Nutrigenomics use gene variants such as FTO, TCF7L2, and PPARG to guide personalised recommendations. These are often combined with biomarkers, such as lipid levels and glucose response, to fine-tune dietary plans [21,22].
- **Gut microbiota:** Differences in microbial composition influence individual glycaemic responses to identical meals. Interventions using fibre, prebiotics, or probiotics are now tailored to enhance microbiome health and metabolic outcomes [23,24,25].
- **Digital tools and behavioural monitoring:** Mobile apps, wearable trackers, and CGM devices provide real-time data. These improve self-awareness, encourage behavioural change, and support better adherence to tailored diets [26,27].
- **Cultural and socioeconomic adaptation:** Success in LMICs depends on affordability, local dietary norms, and food availability. Programmes must use culturally familiar foods and be adapted to local language and literacy levels [28].

Table 1 compares methods used to personalise nutrition. While GI is widely understood and simple, more advanced methods, such as nutrigenomics, provide better precision but require specialised resources.

## 5. Real-World Effectiveness of Personalised Nutrition

Evidence from both clinical trials and real-world implementation studies confirms that personalised dietary strategies can significantly improve outcomes in individuals with T2DM. Low-GI and low-GL diets consistently outperform standard dietary advice by reducing postprandial glucose excursions, improving lipid profiles, and enhancing insulin sensitivity [20,29]. For example, Solomon et al. [30] demonstrated in a real-world intervention that combining a low-GL diet with structured physical activity led to significant reductions in fasting insulin levels and markers of insulin resistance among obese, prediabetic individuals. This combination underscores the practical potential of a low-GL diets as part of comprehensive lifestyle interventions in everyday settings.

In a landmark real-world precision nutrition study, Zeevi et al. [23] used CGM, gut microbiome profiling, and machine learning algorithms to tailor dietary interventions. Their results show significant improvements in postprandial glycaemic responses, even among participants consuming similar meals. This trial highlighted the inadequacy of general dietary guidelines and reinforced the superiority of personalised dietary interventions based on individual biological responses. Digital health tools have also shown effectiveness in practical contexts. For instance, Ehrhardt and Al Zaghal [27] documented how CGM-linked dietary coaching enabled individuals with T2DM to better understand their glycaemic responses to specific foods, reinforcing behavioural changes over time. These tools provide immediate feedback, improving adherence to personalised plans and enhancing self-management capabilities.

Moreover, long-term use of digital PN platforms demonstrated broader metabolic benefits. A review by Barrea et al. [31] reported that these interventions not only improved glycaemic control, but also positively impacted weight management, lipid metabolism, and inflammatory markers. These improvements are critical for reducing the risk of T2DM-related complications, including cardiovascular disease and diabetic neuropathy.

Collectively, these real-world studies illustrate how personalised nutrition strategies, whether through dietary adjustments, biological profiling, or digital coaching can be effectively implemented beyond controlled trial settings. They highlight the practicality, scalability, and clinical relevance of PN interventions in everyday diabetes care.

## 6. Mobile Apps for Diabetes Self-Management

Beyond clinical and provider-focused technologies, mobile health applications are increasingly being developed to empower individuals to manage type 2 diabetes through self-monitoring, education, and behaviour change. These apps support personalised care by offering tools such as meal planning, glucose tracking, physical activity monitoring, and goal setting—fostering greater autonomy and engagement in diabetes self-management.

Popular consumer-facing applications, such as Klinio, MyFitnessPal, and Glucose Buddy, exemplify this approach. Klinio provides customised meal plans tailored to users’ dietary preferences, glycaemic targets, and physical activity levels, making it a robust tool for managing diabetes [32]. MyFitnessPal enables users to log meals, monitor macronutrient intake, and sync data with fitness devices, promoting better awareness of dietary patterns [33]. Glucose Buddy allows for comprehensive tracking of blood glucose readings, medication usage, and carbohydrate intake, supporting daily management decisions [34].

These platforms are generally accessible and cost-effective, helping users recognise behavioural patterns and align their routines with glycaemic control goals. Many also incorporate features such as gamification and social support to enhance user engagement and long-term adherence. Although most of these tools are developed for high-income settings, they offer considerable potential for adaptation in LMICs. Customising app content to reflect local languages, culturally relevant foods, and offline functionality can significantly improve usability in underserved areas. Ensuring compatibility with basic smartphones and incorporating culturally sensitive dietary guidance can further expand their reach.

Ultimately, these mobile applications facilitate a more participatory model of care, shifting part of the responsibility for diabetes management from healthcare providers to individuals and families. Nevertheless, rigorous evaluation of user experience, data accuracy, and long-term health outcomes remains essential to ensure their safe, effective, and equitable integration into healthcare systems.

## 7. Behavioural and Cultural Considerations in Personalised Nutrition for T2DM

The success of personalised nutrition in managing T2DM depends on more than scientific accuracy. It must reflect individual behaviours and cultural contexts to be truly effective. Eating habits are shaped by a complex interplay of social norms, traditions, food availability, and economic status. In many African settings, including rural and peri-urban areas of South Africa, a larger body size is traditionally perceived as a sign of wealth, strength, and good health. This belief remains widespread and can limit individual motivation to engage in weight-reduction interventions or adopt lower-calorie diets [35,36].

Gender dynamics also play a critical role in shaping dietary behaviours. Women, often responsible for food preparation and family care, may prioritise the needs of others over their own health. Men, on the other hand, may resist engaging with healthcare services due to social stigma, traditional masculinity norms, or fear of judgement [37].

Traditional diets, which centre on high-GI foods such as pap (maize porridge), fried meats, and refined carbohydrates, remain deeply embedded in household routines. Replacing these with healthier alternatives proves difficult due to cultural attachment, taste preferences, and limited affordability [38]. In many rural areas, poverty further restricts access to nutrient-dense options such as fruits, vegetables, and lean proteins, exacerbating nutrition-related inequalities [39].

Religious and communal practices, including fasting and ritual feasts, also shape food choices and often override clinical dietary recommendations. Such practices may lead to inconsistent adherence to personalised diet plans, particularly during religious festivals or community gatherings [40].

To improve the effectiveness of personalised nutrition, interventions must include culturally adapted counselling, community-based education, and practical dietary tools such as visual guides in local languages. Using familiar ingredients and recipes in nutritional education enhances relevance and understanding. Engaging family members, local leaders, and healthcare providers ensures support systems that reinforce healthy choices. These community-centred, culturally sensitive approaches build trust, encourage dietary adherence, and facilitate long-term behavioural change, which is essential for improved diabetes outcomes.

## 8. Implementation Challenges of Personalised Nutrition in T2DM

Despite the potential of personalised nutrition, its practical application in T2DM care remains limited in LMICs, including South Africa [41].

**Figure 1:**
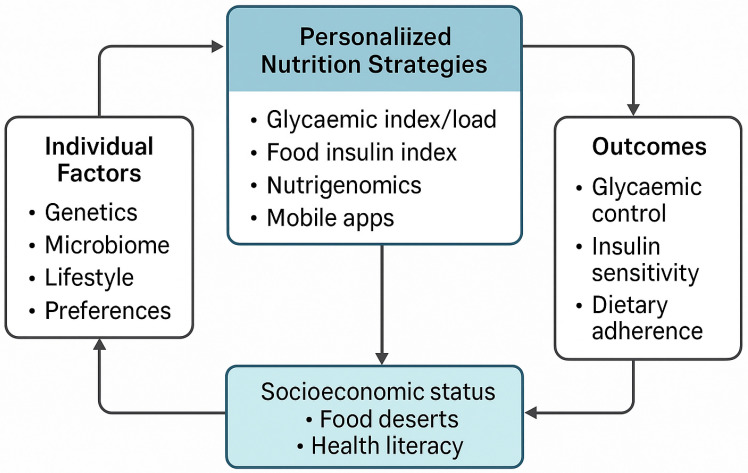
Conceptual framework of personalised nutrition for T2DM.

This framework illustrates how individual genetic, metabolic, behavioural, and environmental factors shape dietary counselling (Figure 1). These are supported by culturally adapted education and digital tools.

### 8.1. Implementation Barriers

#### 8.1.1. Healthcare Infrastructure and Human Resources

There is a significant shortage of qualified nutritionists and registered dietitians in both urban and rural areas, particularly in LMICs, limiting the availability of expert nutritional guidance in primary healthcare settings. In many rural communities, care often relies on community health workers (CHWs), who typically lack specialised training in delivering personalised nutrition counselling [42,43]. As a result, they may struggle to translate complex nutritional concepts into practical, individualised advice for patients. In addition to human resource constraints, many healthcare facilities are under-resourced, further hindering the effective implementation of personalised nutrition strategies. Essential diagnostic tools, such as CGMs, lipid profiling equipment, and microbiome assessment technologies, are frequently unavailable or prohibitively expensive in low-resource settings [23,44]. This lack of infrastructure compromises the early detection, monitoring, and ongoing management of T2DM, ultimately limiting the impact of tailored nutritional interventions.

#### 8.1.2. Digital Divide

The high price of CGMs, smartphones, and internet-dependent applications restricts access among socioeconomically disadvantaged populations [45,46]. Beyond the high costs, many individuals in LMICs face structural limitations such as food deserts—geographical areas with limited access to affordable, healthy foods. In such settings, even the best-tailored dietary guidance may be impractical if individuals cannot access or afford recommended food items. This lack of alignment between PN advice and local food availability compromises both efficacy and adherence.

Moreover, reliable internet connectivity and digital literacy are essential for using these platforms remain limited in many underserved regions. Many PN platforms are designed for high-income, English-speaking users, with limited incorporation of local languages, food items, or dietary customs, thereby reducing both usability and engagement in LMIC settings [47]. This creates a digital divide that risks exacerbating existing health inequities, as populations most burdened by T2DM may be the least equipped to benefit from technology-based interventions. Developing affordable, culturally appropriate digital tools is essential for equitable personalised nutrition. Collaboration between policymakers and tech developers can help make these innovations accessible to all populations.

#### 8.1.3. Health Literacy

Low health literacy among patients, coupled with complex nutritional terminology, often hinders understanding and adherence to personalised dietary recommendations. Terms such as insulin response, GI, GL, and FII are frequently unfamiliar or difficult to grasp [48]. At the same time, healthcare providers in LMICs may lack adequate training to interpret and apply data from advanced tools such as microbiome-guided interventions and GL-based planning [8]. The absence of culturally appropriate educational materials and simplified language further reduces patient engagement, limiting the effectiveness of personalised nutrition strategies.

#### 8.1.4. Policy and Sustainability Gaps

Personalised nutrition interventions are mostly limited to academic or pilot projects. They have not yet been widely adopted into national healthcare systems or funded through public health budgets [6]. In most LMICs, nutrition services are seen as supplementary rather than essential, especially in rural clinics where medical priorities often focus on infectious diseases or maternal health [49]. This lack of integration weakens long-term sustainability and limits the reach of personalised interventions. For meaningful impact, nutrition care must be recognised as a core component of chronic disease management and supported by consistent funding, policy inclusion, and healthcare workforce development.

Table 2 summarises key obstacles to implementing personalised nutrition in LMICs. These include limited staff, unaffordable technologies, low patient understanding, and poor infrastructure. Solutions must include system-level reforms and community engagement to close the gap between innovation and everyday healthcare delivery.

## 9. Integrating Personalised Nutrition into Primary Care for T2DM Management in LMICs

Personalised nutrition must become a core part of primary healthcare (PHC) if it is to serve as a sustainable strategy for managing and preventing T2DM in LMICs. PHC systems are the main entry point for most individuals and offer an ideal platform for early detection, health education, and chronic disease management [43,50]. Embedding dietary counselling into PHC helps bridge gaps in access, affordability, and cultural relevance, particularly for marginalised populations.

### 9.1. Task-Shifting and Capacity Building

Task-shifting is a World Health Organization-endorsed strategy that reallocates duties from specialists to trained CHWs, nurses, or counsellors. This model has shown success in expanding healthcare coverage in low-resource environments [51]. With targeted training, CHWs can offer basic dietary guidance using tools, such as the GI and GL, tailored to locally available and culturally appropriate foods [52]. This decentralised approach builds local capacity and ensures that nutrition services are community-centred and sustainable.

Training programmes should also include behavioural counselling skills, enabling CHWs to deliver messages in an engaging and supportive way. Ongoing supervision, refresher courses, and mentorship systems are vital for maintaining quality and confidence among frontline workers. Involving PHC staff at all levels ensures shared responsibility for nutrition interventions and encourages an integrated, team-based approach to diabetes care.

### 9.2. Context-Specific, Standardised Tools

Effective personalised nutrition must be aligned with local dietary patterns, beliefs, and economic constraints. Standardised tools that incorporate familiar foods, traditional meals, and culturally sensitive messaging are more likely to be understood and adopted. Studies have shown that food-based dietary guidelines that use local visuals and examples significantly improve comprehension and adherence [35].

In South Africa, a family-centred nutrition and physical activity programme in Limpopo successfully engaged households through the use of common staples and practical meal planning [53]. Family involvement helps build support systems and reinforces consistent dietary habits.

Key tools to enhance this approach include the following:

- Visual food charts with staples such as maize, samp, legumes, and sweet potatoes;
- Educational leaflets written in local languages with illustrations;
- Real-life examples and modified traditional recipes discussed during counselling sessions.

These context-specific materials help demystify nutrition concepts and make dietary change more achievable for individuals and families. Tailoring tools to the social and economic realities of communities strengthens engagement and supports sustainable health improvements.

### 9.3. Policy Integration for Sustainability

Sustainable impact depends on integrating personalised nutrition into national health systems and policies, especially NCD strategies. Policy alignment should include routine nutrition screening during PHC visits, development of national dietary guidelines, and inclusion of culturally appropriate dietary advice in clinical protocols [54].

Public health messaging must reflect local customs and values to ensure community buy-in. Brazil’s Family Health Strategy serves as a leading example, demonstrating how PHC-based nutrition interventions can reduce metabolic risk factors and improve health outcomes over time [55]. Involving families in health planning and connecting them with local PHC teams enhances accountability and deepens the impact of interventions [56].

Strong policy support also enables resource allocation, professional training, and integration of personalised nutrition into electronic health records and monitoring systems. This ensures consistent implementation and builds a foundation for evaluation and refinement.

### 9.4. Leveraging Technology in PHC

Digital innovations offer scalable solutions for delivering personalised nutrition, especially in rural or resource-poor regions. Mobile health tools help patients stay engaged, track their progress, and receive reminders tailored to their dietary needs and preferences [57].

Examples of promising tools include:

- Text message campaigns providing localised dietary advice in preferred languages;
- Mobile apps used by PHC workers to support point-of-care counselling;
- Continuous glucose monitors and wearables linked to clinic data for ongoing feedback.

Local adaptation is key. Technology should account for literacy levels, language diversity, and digital access. Training for healthcare workers and end-users ensures usability and maximises impact. When integrated into PHC systems and supported by trained staff, digital tools enhance adherence, enable remote monitoring, and contribute to improved glycaemic control and patient empowerment [45].

## 10. Future Directions and Research Needs

In LMICs, financial and workforce constraints call for cost-effective strategies to support the sustainable adoption of personalised nutrition [58]. Despite its potential, PN still requires more evidence to support integration into routine diabetes care. Key areas needing investigation include feasibility, equity, long-term effectiveness, and scalability. Research should also explore whether PN can reduce healthcare costs through prevention of complications such as cardiovascular disease and kidney failure, both common among patients with poorly controlled T2DM [43,59].

### 10.1. Evaluating Long-Term (Above 12 Months) Outcomes in Real-World Settings

Most current findings are based on short-term trials (<12 months) in controlled environments. There is a clear need for longitudinal research in real-world contexts, particularly in LMICs. Future studies should measure the ongoing effects of PN on glycaemic control, body weight, treatment adherence, and quality of life. These studies should also consider the influence of food insecurity, gender roles, and family decision-making on intervention success [23].

### 10.2. Cost-Effectiveness and Scalability

Wider uptake of PN will depend on its cost-effectiveness. Research should examine low-cost delivery models such as task-shifting to community health workers. Comparative economic analyses should evaluate whether PN delivered via primary care leads to cost savings through fewer complications and hospital admissions when compared to standard diabetes care [43].

### 10.3. Gender-Sensitive and Culturally Tailored Interventions

Cultural norms and gender dynamics strongly influence food access and dietary practices. In some contexts, women may have limited control over household food choices due to cultural expectations or financial dependence [59]. Future interventions should be co-designed with local communities to ensure cultural relevance, inclusion, and gender sensitivity. This collaborative approach increases both engagement and effectiveness.

### 10.4. Digital Integration for Wider Access

Digital platforms offer an opportunity to expand access to PN. Tools such as electronic health records, mobile apps, and SMS-based systems can reach broader populations, especially younger people and those in remote areas. However, future work must also address challenges such as digital literacy, data protection, and disparities in internet access to avoid worsening health inequalities [41].

## 11. Conclusions

Personalised nutrition marks a paradigm shift from generic dietary recommendations to tailored interventions grounded in individual biology, lifestyle, and socio-cultural context. Evidence increasingly supports its potential to improve glycaemic control, enhance long-term dietary adherence, and empower individuals with type 2 diabetes to actively manage their condition. PN offers added value over conventional dietary approaches by incorporating genetic, metabolic, and behavioural data, allowing for more precise and sustainable interventions.

However, translating this potential into routine practice, particularly in LMICs, remains complex. Key barriers include limited access to trained nutrition professionals, high costs of assessment tools such as CGMs or genotyping platforms, and the need for culturally relevant dietary frameworks. To address these, a phased implementation strategy could be considered, beginning with pilot programs integrated into existing diabetes care models and supported by community health workers and digital decision-support tools. These early-stage programs can provide valuable insights into feasibility, scalability, and acceptability.

To unlock the full benefits of PN, a coordinated effort is required. Health systems, researchers, policy-makers, and technology developers must collaborate to co-create scalable, affordable, and context-sensitive PN models. Investment in implementation research is essential to assess real-world outcomes, cost-effectiveness, and strategies for integration into primary care.

## 12. Limitations

This narrative review provides important insights into PN in the context of type 2 diabetes, yet several limitations must be acknowledged. As a narrative review, the methodology is inherently interpretative and does not employ the structured protocols or formal quality appraisal processes typical of systematic reviews, which may introduce selection and synthesis bias.

A key limitation is the considerable heterogeneity across the included studies. Definitions and outcome measures, such as glycaemic control, adherence, and metabolic markers, varied widely, making it difficult to compare findings directly or draw robust conclusions. The inconsistency in outcome definitions could affect the interpretation and generalisability of the evidence, particularly when assessing the impact of different dietary strategies such as GI, GL, and FII.

There is also no universally accepted definition of “personalised nutrition,” leading to variability in its conceptualisation and operationalisation across studies. This lack of standardisation complicates efforts to synthesise findings and develop unified guidelines for implementation.

The geographical distribution of the literature presents another challenge. The majority of studies were conducted in high-income countries, where advanced diagnostic tools and digital health infrastructure are more readily available. There is a critical shortage of data from lower-income areas, limiting the applicability of findings in settings where resources, dietary patterns, and healthcare access differ significantly. Future research in LMICs should be supported through targeted funding, international collaborations, and capacity building to adapt PN tools to local contexts.

Additionally, many studies focused on short-term outcomes, leaving gaps in understanding the long-term effects of PN on diabetes complications, healthcare costs, and quality of life. The psychosocial and behavioural aspects of dietary adherence, which are critical for real-world impact were also underexplored.

Lastly, although this review touches on emerging tools such as CGM, microbiome analysis, and mHealth technologies, many of these innovations remain in early development stages. Their limited validation, high cost, and lack of availability in LMICs further constrain their current utility in global diabetes care.

## Figures and Tables

**Table 1:** Overview of key personalised nutrition strategies in T2DM.

Strategy	Description	Strengths	Limitations
Glycaemic index	Ranks foods by glucose impact	Simple, practical	Ignores portion sizes
Glycaemic load	Considers GI and quantity	Better prediction of glucose levels	Slightly more complex for patients
Food insulin index	Assesses insulin response from all macros	Captures full insulin dynamics	Limited data, less familiar
Nutrigenomics	Tailors diet using genetic markers	Highly individualised	Expensive and less accessible

**Table 2:** Barriers to implementing personalised nutrition in LMICs.

Barrier	Description	Suggested Solutions
Lack of trained staff	Too few dietitians in clinics and PHC	Train and support CHWs
Digital divide	CGMs and apps are expensive and limited	Government subsidies, partnerships
Health iliteracy	Complex terms confuse many patients	Simplify materials; adapt to local contexts
Healthcare infrastructure	Limited access to diagnostic tools	Use mobile-friendly or basic diagnostic tools
Policy and sustainability gaps	PN is not integrated into national health policies; nutrition is often sidelined in PHC	Embed PN into NCD strategies and PHC protocols; ensure dedicated funding
